# The Relationship between Cell Number, Division Behavior and Developmental Potential of Cleavage Stage Human Embryos: A Time-Lapse Study

**DOI:** 10.1371/journal.pone.0153697

**Published:** 2016-04-14

**Authors:** Xiangyi Kong, Shuting Yang, Fei Gong, Changfu Lu, Shuoping Zhang, Guangxiu Lu, Ge Lin

**Affiliations:** 1 Institute of Reproductive and Stem Cell Engineering, college of basic medicine, Central South University, Changsha, China; 2 Key Laboratory of Reproductive and Stem Cell Engineering, Ministry of Health, Changsha, China; 3 Reproductive and Genetic Hospital of CITIC-Xiangya, Changsha, China; 4 National Engineering and Research Center of Human Stem Cells, Changsha, China; Peking University Third Hospital, CHINA

## Abstract

Day 3 cleavage embryo transfer is routine in many assisted reproductive technology centers today. Embryos are usually selected according to cell number, cell symmetry and fragmentation for transfer. Many studies have showed the relationship between cell number and embryo developmental potential. However, there is limited understanding of embryo division behavior and their association with embryo cell number and developmental potential. A retrospective and observational study was conducted to investigate how different division behaviors affect cell number and developmental potential of day 3 embryos by time-lapse imaging. Based on cell number at day 3, the embryos (from 104 IVF/intracytoplasmic sperm injection (ICSI) treatment cycles, n = 799) were classified as follows: less than 5 cells (< 5C; n = 111); 5–6 cells (5–6C; n = 97); 7–8 cells (7–8C; n = 442), 9–10 cells (9–10C; n = 107) and more than 10 cells (>10C; n = 42). Division behavior, morphokinetic parameters and blastocyst formation rate were analyzed in 5 groups of day 3 embryos with different cell numbers. In <5C and 5–6C embryos, fragmentation (FR; 62.2% and 30.9%, respectively) was the main cause for low cell number. The majority of 7–8C embryos exhibited obvious normal behaviors (NB; 85.7%) during development. However, the incidence of DC in 9–10C and >10C embryos increased compared to 7–8C embryos (45.8%, 33.3% vs. 11.1%, respectively). In ≥5C embryos, FR and DC significantly reduced developmental potential, whereas <5C embryos showed little potential irrespective of division behaviors. In NB embryos, the blastocyst formation rate increased with cell number from 7.4% (<5C) to 89.3% (>10C). In NB embryos, the cell cycle elongation or shortening was the main cause for abnormally low or high cell number, respectively. After excluding embryos with abnormal division behaviors, the developmental potential, implantation rate and live birth rate of day 3 embryos increased with cell number.

## Introduction

In current in vitro fertilization (IVF) practice, day 3 (d3) cleavage embryo transfer is routine in many assisted reproductive technology centers. To achieve satisfactory pregnancy outcomes, embryos are usually selected according to standardized scoring criteria for transfer; typically determined by cell number, cell symmetry and fragmentation [1]. Cell number is the most critical indicator for development potential, as it can directly reflect an embryo’s ability for cell cycle progression. It is generally accepted that d3 human embryos with good developmental potential should develop to the 7–8 cell stage [1]. Studies have shown that embryos with either lower or higher cell numbers have significantly reduced developmental potential[2–4]. Furthermore, it has also been reported that the proportion of blastocysts that appeared to be normal was significantly higher among d3 embryos with 7–9 cells (41.9%) compared to embryos with less than 7 cells (13.8%) or more than 9 cells (27.5%)[3]. This phenomenon was also evident in embryos with low cell numbers, whereby transferring 4-cell embryos resulted in a significantly higher implantation rate (23%) than transferring of 2-cell (12%) or 3-cell embryos (7%) in d2[2]. It has been indicated that embryos with lower cell numbers experience more fragmentation, where mean blastomere size decreased significantly with increasing degree of embryonic fragmentation, and highly fragmented embryos showed a 43–67% reduction in blastomere volume compared with embryos with no fragmentation [5]. The release of large fragments at an early stage may deplete the embryo of essential organelles and structures such as mitochondria and pinocytotic caveolae, which are involved in the uptake of exogenous proteins, and may lead to growth arrest [6]. Other human studies have been seemingly contradictory, suggesting that embryos with high cell numbers form the highly desired, good quality blastocysts (4AA or better) with the greatest clinical potential, in comparison to the other d3 embryo cleavage groups [7–9].

In recent years, with the aid of time-lapse technologies, objective and accurate information, such as timing of development and division behavior, can be recorded and annotated. Blastocyst formation [10], blastocyst quality [11], implantation [12–14] and live birth [15] can be predicted by specific time-lapse parameters. A number of studies have reported that certain division behaviors, such as direct division from 1 cell to ≥3 cells, can influence growth rate and decrease blastocyst formation and implantation [12, 16, 17]. Time-lapse studies have consistently indicated that embryos that cleave at intermediate time-points have significantly improved chance of implantation, when compared with embryos that have either developed faster or slower. Furthermore, embryo viability has also been associated with a tightly regulated sequence of cellular events that begin at the time of fertilization[18]. However, there is limited understanding of embryo division behavior and its association with embryo cell number.

In the current retrospective study, we aim to understand division behavioral characteristics in embryos with different growth rates, identify cell cycle progression patterns in embryos with varying cells numbers, and determine the relationship between growth rate, division behavior and developmental potential.

## Materials and Methods

### Embryo source

A retrospective study was conducted on 799 normal fertilized embryos (from 104 IVF/intracytoplasmic sperm injection (ICSI) treatment cycles) undergoing time-lapse monitoring between 2011 and 2014. This project was approved by the Institutional Review Board of the Reproductive and Genetic Hospital of CITIC Xiangya on 28 February 2012 (reference LL-SC-SG-2012-004). And all the patients have signed the written informed consent approved by the IRB for the procedures.

### Embryo culture and time-lapse recording

All pronuclear stage embryos were placed individually into microwells of a well-of-the-well (WOW) culture dish (9 or 16 microwells; Vitrolife, Budapest, Hungary) and were cultured in sequential media (G1 and G2, Vitrolife; Goteborg, Sweden). All embryos were cultured to the blastocyst stage (~5 days) in a 37°C incubator with a 6% CO2, 5% O2 and 89% N2 atmosphere. Time-lapse images were acquired using a Primo Vision system (Vitrolife, Budapest, Hungary). For culture medium change on d3, the dish was removed from the incubator and all embryos transferred to the same position of another WOW dish containing a 50-μl micro droplet of G2.5 medium covered with oil (Ovoil; Vitrolife, Budapest, Hungary). The dish was then returned to the incubator, placed in the sample holder of the digital microscope, and the time-lapse monitoring was continued. Images of each embryo were recorded every 5 minutes. Division behaviors including fragmentation and direct cleavage, and morphokinetic parameters including cc1 (duration of first cell cycle or mitosis, from completion of 2 cells to insemination), Ecc2 (duration of second embryo cell cycle, from 2 to 4 cells) and Ecc3 (duration of third embryo cell cycle, from 4 to 8 cells) were analyzed. Moreover, t0 means the time of insemination for routine IVF or mid-time of ICSI as previously described[19]. Blastocyst morphology was evaluated at day 5 (d5; 116 ± 2 hours) after insemination, according to Gardner’s scoring system [20]. Blastocysts that were scored at least as 4BB or higher quality, including 4,5,6AA, AB, BA and BB, were defined as good quality blastocysts, and other blastocysts were defined as poor quality blastocysts. Morphokinetic parameters were recorded until embryos finished three cleavage cycles.

### Statistical analysis

Data analysis was performed by using Statistical Package for Social Sciences for Windows, version 18.0 (Chicago, IL, USA). The rates of blastocyst formation were analyzed by partitions of the chi-squared method. Categorical variables were analyzed by a chi-squared test and continuous variables were analyzed by t-test if they followed normal distribution. Data that were not normally distributed were analyzed by Mann-Whitney-Wilcoxon tests. A logistic regression analysis were performed for blastocyst formation rate and good quality blastocyst rate including woman`s age and cell numbers. A P-value <0.05 was considered statistically significant.

## Results

### Division behaviors in low- and high cell number embryos

**Fig 1:**
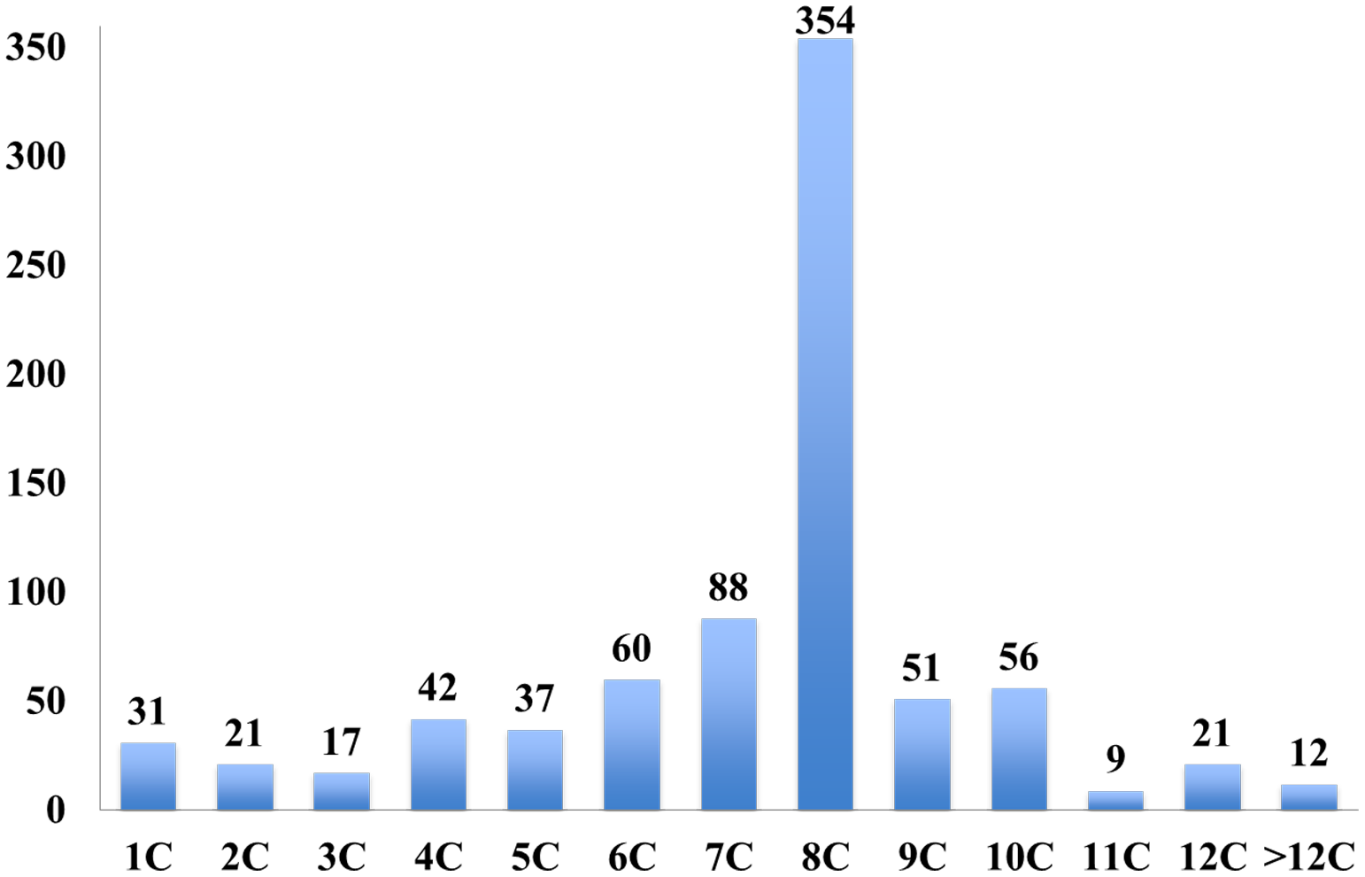
Cell number distribution for all embryos on day 3.

**Fig 2:**
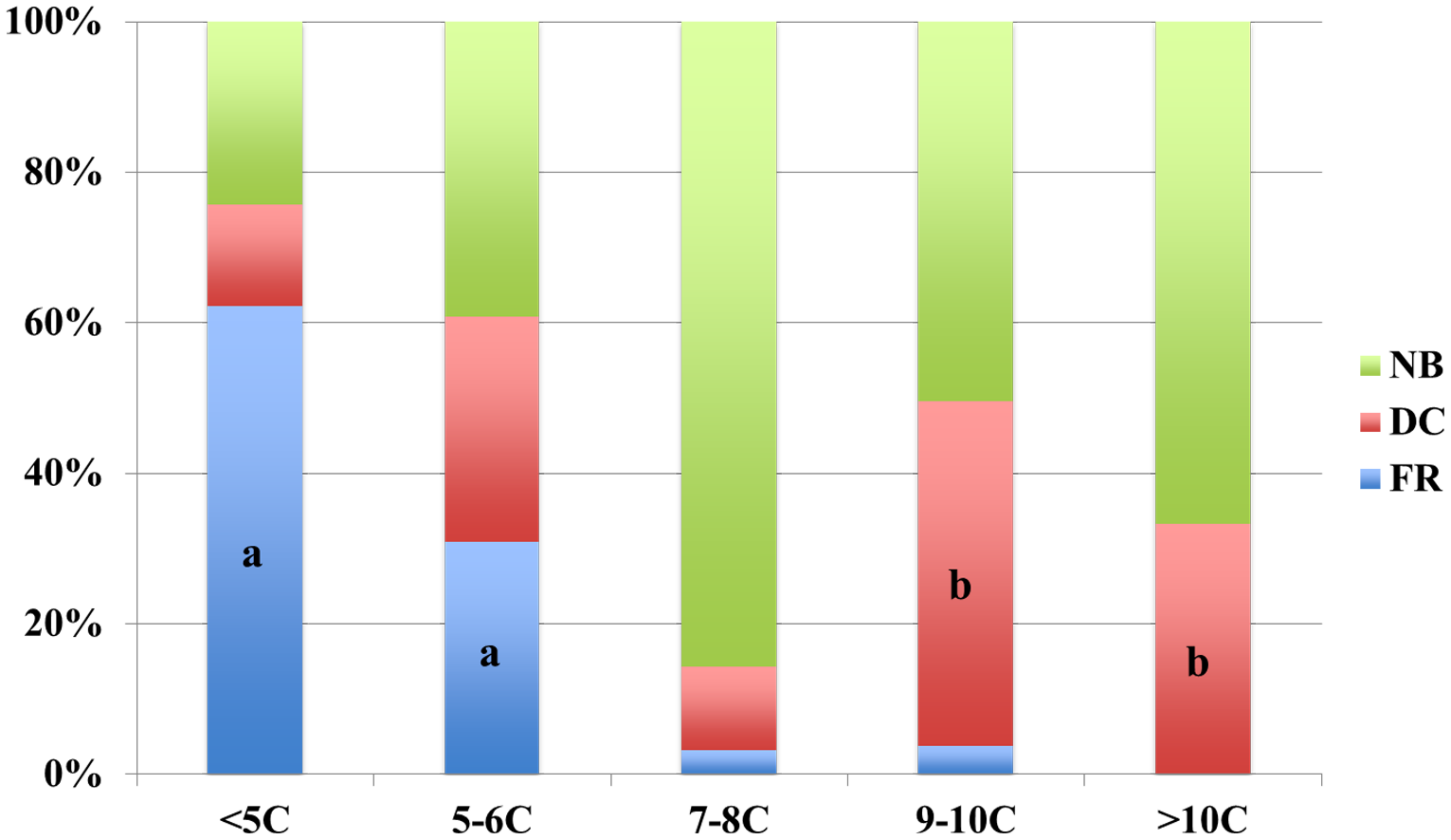
Division behaviors and cell number (n = 799). (A) In <5C and 5–6C groups, FR was the main cause of blastomere loss when compared to other groups (P < 0.01). (B) Comparing to 7–8C embryos, DC became the leading abnormal behavior in 9–10C and >10C groups, P < 0.01 respectively. FR: fragmentation; DC: direct division; NB: normal behavior.

Of the 799 normal fertilized oocytes, the majority (67.5%) had developed to the third cleavage stage on d3 morning. Eight-cell stage embryos were dominant (44.3%), 13.9% of embryos (111) had less than 5 cells, and 18.6% of embryos (149) had cell numbers beyond 8 cells (Fig 1). Based on d3 cell number, all embryos were divided into 5 groups: less than 5 cells (<5C; n = 111); 5–6 cells (5–6C; n = 97); 7–8 cells (7–8C; n = 442), 9–10 cells (9–10C; n = 107) and more than 10 cells (>10C, n = 42). In order to investigate the cause for the low and high cell number embryos, we annotated and analyzed the division behaviors in the time-lapse records. With the cell number decreased, embryos have an increased tendency for the fragmentation (FR), which was defined as a daughter blastomere turning into several fragments after division. In <5C embryos, up to 62.2% of embryos experienced FR, and it was the main reason for blastomere loss and low cell numbers on d3 (Fig 2). The other embryos did not reach the third cleavage stage, because of direct cleavage (DC)-induced or unexplained arrest. In 5–6C embryos, FR (30.9%) was also the leading cause for low cell number. However, DC (29.9%), which was defined as one blastomere dividing into three or more blastomeres, acted as another key cause of growth retardation, resulting in low cell numbers. We observed that in those embryos, 65.5% of the blastomeres that underwent DC, did not enter the next cell cycle. The majority of 7–8C embryos (85.7%) exhibited obvious normal behavior (NB) during development. As expected, the incidence of NB decreased in >8C embryos, and DC became the leading abnormal behavior in the 9–10 C and >10 C groups, compared to 7–8C embryos (45.8% and 33.3% vs. 11.1%, respectively; P < 0.01). All the NB embryos in >9C groups entered the forth cleavage stage, and cell cycle shortening was the most likely cause for high cell numbers.

### Cell number, division behaviors and embryo viability

**Fig 3:**
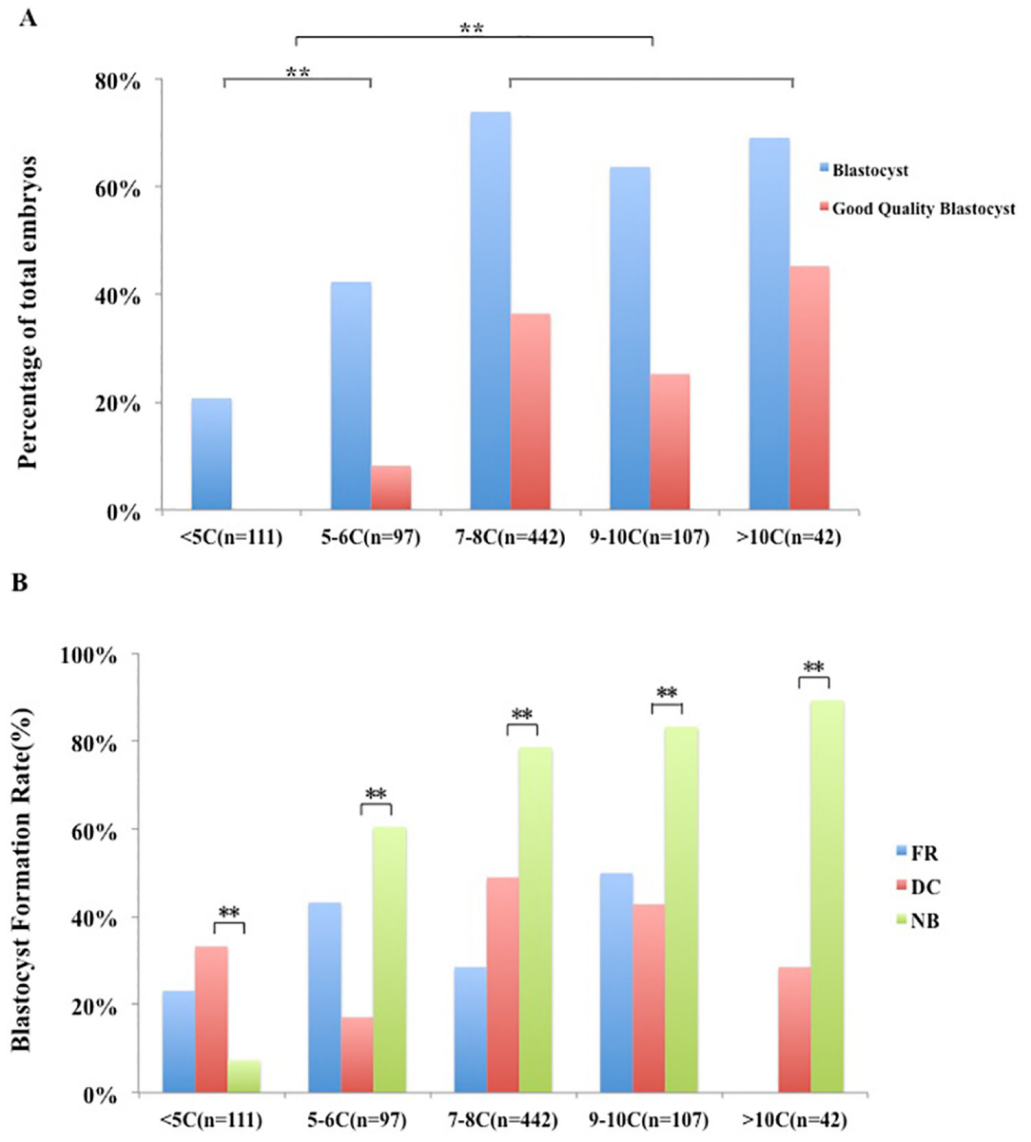
Cell number, cleavage behavior and developmental potential. (A) Cell number and developmental potential. Blastocyst formation rate and good quality blastocyst formation rate increased with cell number, and reached high levels in the 7–8C and >10 C groups. (B) Growth rate, division behaviors and embryo viability. DC decreased blastocyst formation rate in ≥5 C embryos significantly (P < 0.01). In embryos with normal division behavior, embryo development potential increased with cell number (P < 0.01). FR: fragmentation; DC: direct division; NB: normal behavior.

Of the 799 normal fertilized embryos, 61.1% (488) developed to blastocysts on d5 of culture and 26.9% (215) were good quality blastocysts. In embryos with less than 9 cells, blastocyst formation rate and good quality blastocyst formation rate increased with cell number, from 20.7% (23/111) and 0% (0/111) in <5C embryos, respectively, to 73.8% (326/442) and 36.4% (161/442) in 7–8C embryos, respectively (P < 0.01; Fig 3A). The rate of blastocyst development and good quality blastocyst development was at a high level in ≥7C embryos, especially at the 7–8C stage (73.8% and 36.4%, respectively) and >10C stage (69.0% and 45.2%, respectively).

We further investigated the relationship between cell number, division behaviors and embryo viability. In ≥5C embryos, FR and DC influenced blastocyst formation, and DC significantly reduced developmental potential (P < 0.01). In 5–6C embryos, DC showed a worse effect on blastocyst formation when compared to FR (P < 0.05), and both FR and DC reduced developmental potential in 7–8C embryos, but did not reach statistical significance. However, in <5C embryos, no obvious difference of blastocyst formation was observed in NB (7.4%, 2/27), FR (23.2%, 16/69) and DC (33.3%, 5/15) embryos (Fig 3B). In 7–8C embryos, the occurrence of DC was similar in the 1^st^ (34.7%, 17/49), 2^nd^ (32.6%, 16/49) and 3^rd^ (32.6%, 16/49) cleavage. In 9–10C and >10C embryos, DC mainly occurred during the 2^nd^ (57.1%, 28/49, P<0.01) cleavage. In embryos with normal division behavior, we observed a trend in development potential that increased with cell number. In embryos that remained at the 2^nd^ cleavage stage on d3, development potential decreased to 7.4%, and following entry into the 3^rd^ cleavage stage, a higher cell number was associated with a higher blastocyst formation rate (P < 0.05).

Of the 84 patients involved in this study, the median (P25, P75) age was 30(27,36)years old. To exclude the possible effect of advanced maternal age on embryo viability, we performed a logistic regression analysis for blastocyst formation rate and good quality blastocyst formation rate including maternal age and cell number. The results showed that age and cell number were both factors that affect embryos quality. When we adjusted for age, cell number still significantly influenced embryos developmental potential (S1 Table).

### Dynamics and term development potential in low and high cell number NB embryos

**Fig 4:**
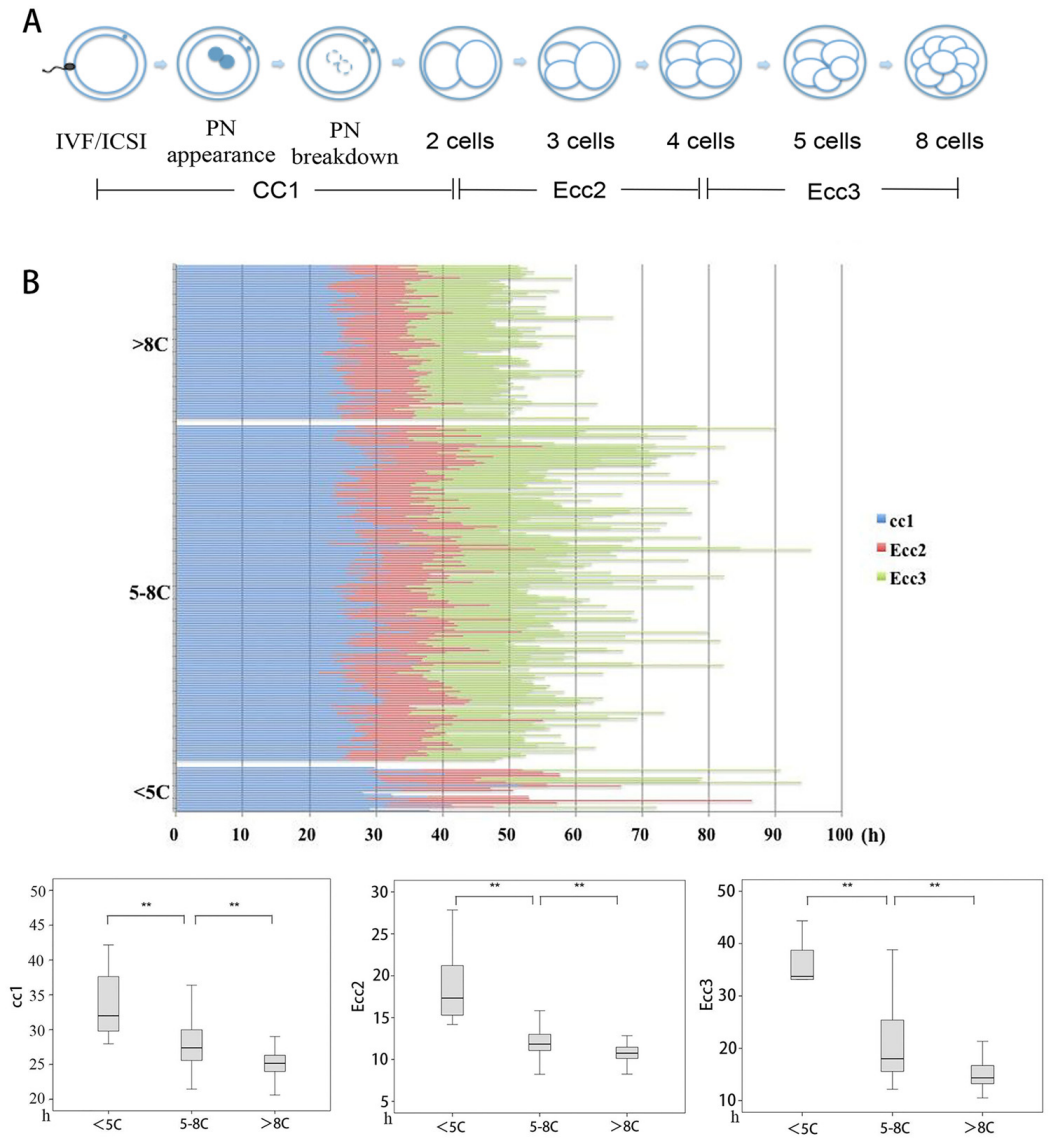
Different morphokinetics parameters in low- and high cell number NB embryos. (A) The first three mitosis cycles of NB embryos from insemination to 8 cells. cc1: duration of first cell cycle or mitosis, from completion of 2 cells to insemination; Ecc2: duration of second embryo cell cycle, from 2 to 4 cells; Ecc3: duration of third embryo cell cycle, from 4 to 8 cells. (B) Different morphokinetics parameters in low- and high cell number NB embryos. <5 C: NB embryos in <5 C embryos; 5− 8C: NB embryos in 5–8C embryos; > 8C: NB embryos in >8 C embryos. **P < 0.01.

Variable cell numbers were observed even in embryos displaying NB. To further investigate the cause of this phenomenon, we compared the duration of each cleavage (cc1, Ecc2, Ecc3) for NB embryos in low- (<5C), intermediate- (5–8C) and high cell number (>8C) embryos (Fig 4A). Since t0 may influence cc1 in our analysis, so we compared cc1 between IVF (n = 119) and ICSI (n = 259) group, we did not found statistics difference between them (27.3(25.7, 30.2) h vs. 27.5 (25.2, 30.8) h, p>0.05). This may indicate no bias exists when we analyzed the duration of the first cell cycle between <5C, 5-8C, and >8C groups. So in the paper, the analysis of cc1 involved both IVF and ICSI embryos. Obvious extension of the duration of cc1, Ecc2 and Ecc3 cleavage cycles were recorded for <5C NB embryos than 5-8C embryos (cc1, 32.0(29.8–37.8) h vs. 27.4(25.6–30.0) h, P<0.01; Ecc2, 17.3(15.3–24.2) h vs. 11.8(11.1–13.0) h, P<0.01; Ecc3, 33.7(28.8–41.5) h vs. 18.0(15.6–25.4) h, P<0.01). Similarly, obvious shortening of the duration of the first, second and third mitosis cycle (P < 0.01) were recorded for >8C NB embryos comparing to the corresponding 5–8C embryos (Fig 4B).

**Fig 5:**
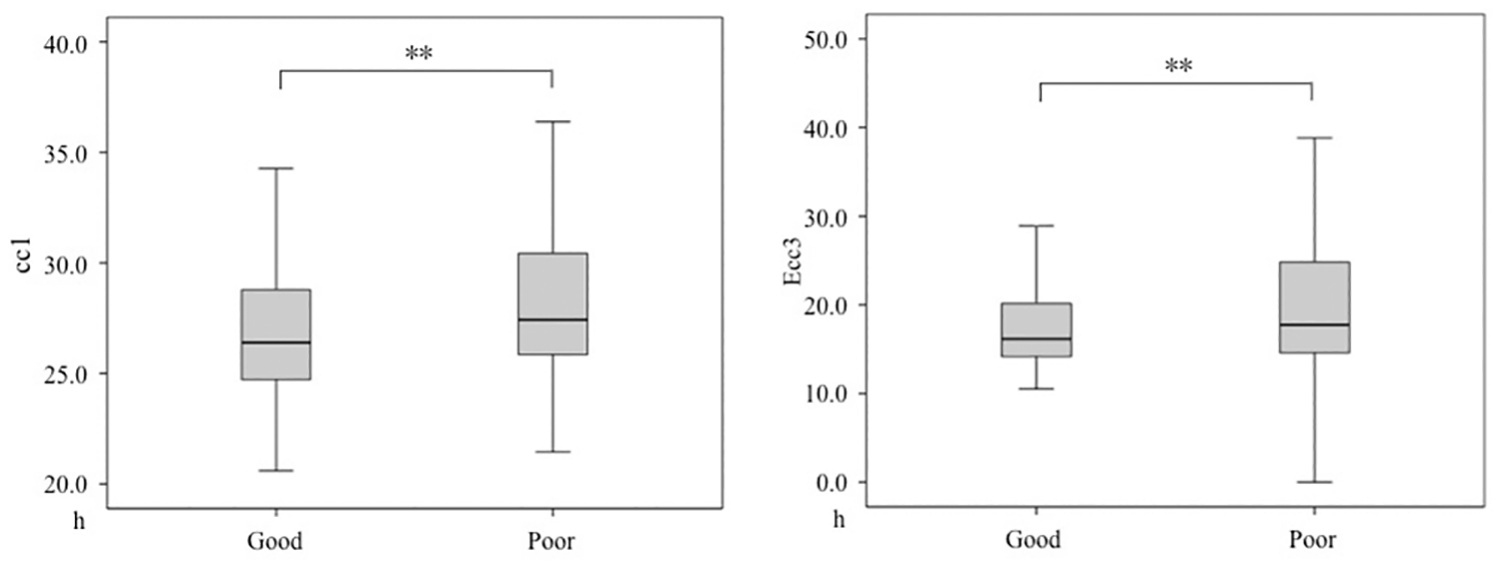
Different morphokinetics parameters in good quality and poor quality blastocysts. Good: Good quality blastocysts; Poor: Poor quality blastocysts. **P < 0.01.

**Fig 6:**
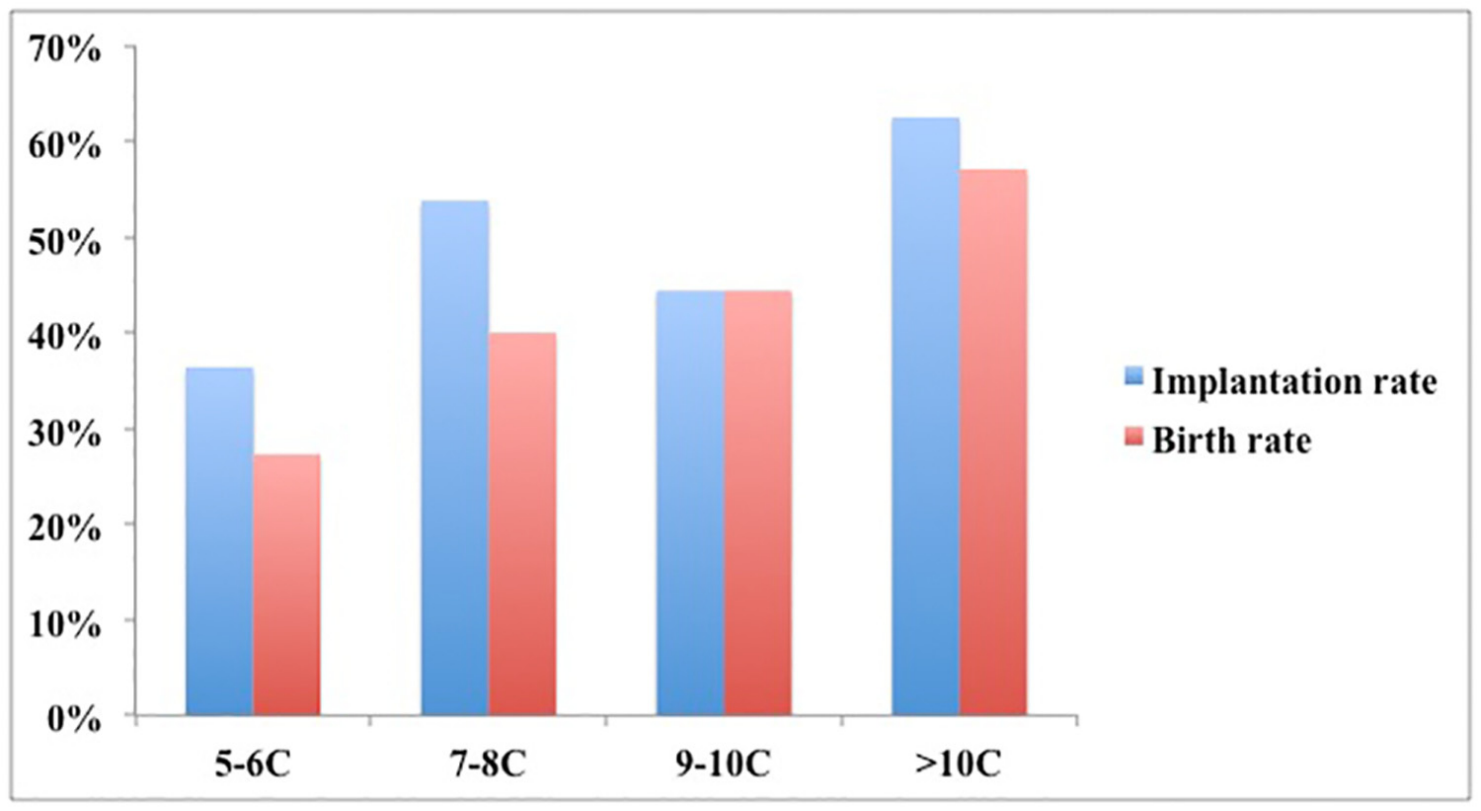
Implantation rate and live birth rate in NB embryos with different cell number.

To investigate whether fast cell cycle progression might correlate with increased developmental potential in NB embryos, we firstly compared the morphokinetics parameters (cc1, Ecc2, Ecc3) between blastocysts with good quality and poor quality. The results showed that the durations of first three divisions (cc1, Ecc2, Ecc3) were longer in the group of good quality blastocysts than other blastocysts, though no statistics significance existed in Ecc2: (cc1, 26.4(24.7, 28.8) h vs. 27.4(25.8,30.5) h, p<0.01;Ecc2, 11.4(10.8,12.5) h vs. 11.7(10.5,12.8) h, p>0.05;Ecc3, 16.2(14.1,20.3) h vs. 17.8(14.6,25.3) h, p<0.01)(Fig 5). So the extension of the duration of first three divisions adversely affected the quality of blastocyst. Then we assessed the relation between developmental dynamics of embryos and their further developmental potential after transfer. In 82 embryos from 63 transfer cycles, only 67 embryos with known implantation data (all transferred embryos either failed to implant (*n* = 33, from 25 cycles) or fully implanted (*n* = 34, from 29 cycles)) were analyzed. The implantation rate and live birth rate had a trend for increasing from 36.4% (5-6C) to 62.5% (>10C) and 27.3%(5-6C) to 57.0%(>10C) with cell number increased in NB embryos (Fig 6).

## Discussion

Growth rate is one of the most important indicators for evaluating embryo quality. With the application of time-lapse imaging technology, embryo cell numbers, timing of development and division behaviors can be evaluated more accurately. In the present study, time-lapse imaging from the pronuclear stage to d5 after insemination provided new insight into the relationship between cell number, division behavior and developmental potential, which cannot be obtained or fully understood without frequent, regular observations. Our results demonstrated that fragmentation and direct cleavage acted as the main reasons for embryos with both low and high cell numbers. After excluding embryos with abnormal division behaviors, development potential, implantation rate and live birth rate increased with cell number.

It has been suggested that variations in growth rate can occur in response to culture conditions, which can affect embryo metabolism. A study by Kasterstein et al. demonstrated that cell number on d3 was higher in 5% O_2_ than in 20% O_2_ [21], and other studies have indicated that culture conditions coupled with paternal effect [22, 23] can affect the duration of the synthesis phase (S-phase) and ooplasm maturity. Results from the current study indicated that fragmentation resulting in fewer surviving blastomeres, DC leading to development arrest and unexplained developmental delays were the main explanations for embryos with low cell numbers. Similarly, DC resulting in surplus blastomeres and faster development timings acted as the main reasons for embryos with high cell numbers. However, our results indicated that DC occurred in embryos of different cell numbers, including low, normal and high cell number embryos. However, DC occurring at different cleavage stages can result in embryos of varying cell numbers [24], and can have different consequences such as developmental arrest or surplus blastomere formation. It has been established that cell cycle duration is approximately 10–12 hours [25]. This interval is sufficient for the embryo to undergo two consecutive phases of cytokinesis and replicate the whole cell genome. A recent investigation by Rubio et al. used a time-lapse monitoring system to detect an abnormally short cell cycle in DC embryos [16], which may result in development arrest or fast growth rate.

Results from the current study indicated that ≥7C embryos showed comparably high development potential, which decreased with cell number in <7C embryos. These results were consistent with previous reports [7], which indicated embryos with ≥7C (and particularly 7–8C embryos) were selected with priority, even without time-lapse monitoring. In our study, fragmentation and direct cleavage significantly reduced developmental potential in ≥5C embryos. Fragmentation can decrease embryo potential by releasing a large quantities of products derived from lysis and degeneration, for example, postlytic DNA fragmentation, which may cause degeneration of adjacent blastomeres or disturb embryo development[26]. In addition, a recent study demonstrated a much lower implantation rate for DC embryos (1.2%) compared to non-direct cleavage embryos (20.2%) [16]. The exact cause of DC remains unclear. Some studies have considered it to be related to the formation of tripolar spindles, caused by abnormally high numbers of centrioles, and driven by excessively penetrating spermatozoa in polyspermic oocytes. In human embryos, tripolar or tetrapolar spindle formation is often associated with abnormal distribution of chromosomes, which can reduce embryo potential [27, 28]. Furthermore, it has also been suggested that high degrees of chromosomal abnormalities are associated with low (<6 cells on d3) or high (>9 cells on d3) cell number embryos [29], which may lead to the failure of compaction and correct activation of the embryonic genome[2, 3], in turn impairing embryo competence. In the current study, <5C embryos have demonstrated little developmental potential regardless of whether or not they displayed abnormal division behaviors. The cause of unexplained developmental delays is still unclear, although one report has associated this phenomenon to egg factor [24].

In the present investigation, development potential increased with cell number after excluding embryos with abnormal division behaviors. In these embryos, cell number and blastocysts quality were both affected by the duration of cell cycle. We hypothesize that embryos with low cell number were the result of prolonged cell cycles, while embryos with high cell number were the result of accelerated cell cycles. In NB embryos, accelerated cell cycle was associated with higher developmental potential; this may indicate that high potential embryos have vigorous growth ability and cell cycle progression could be a latent indicator for embryo selection, although the exact mechanism underlining this phenomenon remains to be investigated.

In the current retrospective study, we have investigated the embryonic behavioral causes of low and high cell number embryos. However, the exact causes of these behaviors and their consequences will require further investigation. Our results have provided important information for embryo selection, which will be beneficial for achieving selective single embryo transfer.

## Supporting Information

S1 Table Logistic regression analysis for blastocyst formation rate and good quality blastocyst formation rate. (DOCX)
