# Supplementary material for: The Relationship between Cell Number, Division Behavior and Developmental Potential of Cleavage Stage Human Embryos: A Time-Lapse Study
Source: PLoS One. 2016 Apr 14;11(4):e0153697. doi: 10.1371/journal.pone.0153697 (PMC4831697; doi:10.1371/journal.pone.0153697)
Supplement: S1 Table — (DOCX) [file pone.0153697.s001.docx]

**S1 Table.** **Logistic regression analysis for blastocyst formation rate and good quality blastocyst formation rate**

| **Factors** | **OR (95%CI)** | ***P* value** |
| --- | --- | --- |
| **Odds of blastocyst formation rate** | | |
| Age (years) |  |  |
| <25 |  | <0.01 |
| 26-30 | 0.74(0.45-1.21) | 0.23 |
| 31-35 | 0.51(0.30-0.88) | 0.02 |
| >36 | 0.22(0.13-0.40) | <0.01 |
| Cell number |  |  |
| >10cells |  | <0.01 |
| 9-10cells | 0.68(0.30-1.55) | 0.36 |
| 7-8cells | 1.02(0.49-2.13) | 0.96 |
| 5-6cells | 0.20(0.09-0.47) | <0.01 |
| <5cells | 0.75(0.03-0.18) | <0.01 |
| **Odds of good quality blastocyst formation rate** | | |
| Age (years) |  |  |
| <25 |  | <0.01 |
| 26-30 | 0.58(0.36-0.93) | 0.03 |
| 31-35 | 0.46(0.26-0.81) | <0.01 |
| >36 | 0.38(0.20-0.69) | <0.01 |
| Cell number |  |  |
| >10cells |  | <0.01 |
| 9-10cells | 0.34(0.16-0.76) | 0.01 |
| 7-8cells | 0.56(0.29-1.10) | 0.09 |
| 5-6cells | 0.06(0.02-0.20) | <0.01 |
| <5cells | 0.00 | 0.99 |

Note: CI = confidence interval; OR = odds ratio
